# Effect of Co-Doping on Cu/CaO Catalysts for Selective Furfural Hydrogenation into Furfuryl Alcohol

**DOI:** 10.3390/nano12091578

**Published:** 2022-05-06

**Authors:** Munsuree Kalong, Sakhon Ratchahat, Pongtanawat Khemthong, Suttichai Assabumrungrat, Atthapon Srifa

**Affiliations:** 1 Department of Chemical Engineering, Faculty of Engineering, Mahidol University, Nakhon Pathom 73170, Thailand; kalong.munsuree@gmail.com (M.K.); sakhon.rat@mahidol.edu (S.R.); 2 National Nanotechnology Center (NANOTEC), National Science and Technology Development Agency (NSTDA), Pathum Thani 12120, Thailand; pongtanawat@nanotec.or.th; 3 Center of Excellence in Catalysis and Catalytic Reaction Engineering, Department of Chemical Engineering, Faculty of Engineering, Chulalongkorn University, Bangkok 10330, Thailand; suttichai.a@chula.ac.th; 4 Bio-Circular-Green-Economy Technology & Engineering Center (BCGeTEC), Department of Chemical Engineering, Faculty of Engineering, Chulalongkorn University, Bangkok 10330, Thailand

**Keywords:** hydrogenation, furfural, furfuryl alcohol, cobalt, copper, calcium oxide

## Abstract

Cu/CaO catalysts with fine-tuned Co-doping for excellent catalytic performance of furfural (FAL) hydrogenation to furfuryl alcohol (FOL) were synthesized by a facile wetness impregnation method. The optimal Co_1.40_Cu_1_/CaO catalyst, with a Co to Cu mole ratio of 1.40:1, exhibited a 100% FAL conversion with a FOL yield of 98.9% at 100 °C and 20 bar H_2_ pressure after 4 h. As gained from catalyst characterizations, Co addition could facilitate the reducibility of the CoCu system. Metallic Cu, Co-Cu alloys, and oxide species with CaO, acting as the major active components for the reaction, were formed after reduction at 500 °C. Additionally, this combination of Co and Cu elements could result in an improvement of catalyst textures when compared with the bare CaO. Smaller catalyst particles were formed after the addition of Co into Cu species. It was found that the addition of Co to Cu on the CaO support could fine-tune the appropriate acidic and basic sites to boost the FOL yield and selectivity with suppression of undesired products. These observations could confirm that the high efficiency and selectivity are mainly attributed to the synergistic effect between the catalytically active Co-Cu species and the CaO basic sites. Additionally, the FAL conversion and FOL yield insignificantly changed throughout the third consecutive run, confirming a high stability of the developed Co_1.40_Cu_1_/CaO catalyst.

## 1. Introduction

An application of the biorefinery concept for biobased chemical and fuel production from agricultural bioresources has been recognized as a sustainable platform process to replace petroleum and petrochemical products [1,2,3,4,5,6,7,8]. Biobased furfural (FAL), derived from lignocellulosic biomass, is a versatile chemical that can be converted into furfuryl alcohol (FOL), tetrahydrofurfuryl alcohol (THFA), 2-methylfuran (2-MF), 2-methyltetrahydrofuran (2-MTHF), furfuryl ethyl ether (FEE), pentanediols (PeD), and others by several thermo-conversion processes such as hydrogenation, hydrogenolysis, and esterification with alcohols [1,3,9,10,11,12,13,14,15,16,17,18,19,20]. Between them, global FAL consumption for FOL production has increased by up to ~65% due to its broad applications for polymeric resins, pharmaceuticals, reactive solvents, and lubricants [21,22,23,24,25,26]. FAL hydrogenation into FOL occurs by H_2_ substitution to an unsaturated C=O bond outside the furan ring, where the reaction rate is faster than the rate for C=O scission and C=C hydrogenation inside the furan ring [27,28,29,30]. However, controlling the selectivity to the FOL product is difficult at a high conversion level, since the target FOL undergoes conversion into undesired products [21,31,32].

Cu-containing catalysts have been suggested as suitable catalysts for FOL production due to their strong interaction with C=O outside the furan ring and weak hydrogenation ability to the C=C bond in the furan ring [33]. An industrial Cu-Cr catalyst has been commercially utilized for the FAL hydrogenation process with high activity, selectivity, and stability for FOL production; nevertheless, toxicity is the major drawback of the catalyst, as it causes environmental impacts and leads to health concerns [34]. Various non-noble heterogeneous catalysts in mono-, di-, and trimetallic components, such as Ni, Co, Cu, Co_2_P, Cu-Ni, Cu-Co, Na-Cu, Cu-Zn, Zn-Co, and NiCoZn supported on Al_2_O_3_, SiO_2_, metal silicate, titanium silicate, zeolitic imidazolate framework (ZIF), carbons, and others, have been recently employed for FAL hydrogenation into FOL [21,31,34,35,36,37,38,39,40,41,42,43,44]. The high catalytic activity and selectivity for FOL production for almost all catalysts have been noticed at a temperature higher than 150 °C under pressurized H_2_. However, a Na–Cu@TS-1 catalyst, namely, a nanosized Cu-supported Na-exchanged titanium silicalite-1 zeolite, has been developed, and 93% FAL conversion with a 91.2 yield of FOL has been obtained at a temperature of 110 °C and H_2_ pressure of 10 bar after 2 h [31]. The enhancement of catalytic activity and selectivity is due to electronic interactions between nanosized Cu and Ti and proper acid/base sites by electron donors of Na species [31]. In addition, the titanium silicalite is well known to have a weak acidity [45]. Moreover, porous metal silicate materials (PMS–2) incorporated by Co/Cu components exhibit a 99% FAL conversion with a FOL yield of 98% at 110 °C and 10 bar H_2_ pressure after 1 h of reaction time, due to their synergistic effects of active Co-Cu species dispersed in the PMS structure [35]. Accordingly, catalyst structure modifications, electronic interactions, and tunable acid and base sites have been stated to be significant catalyst properties for high selectivity to FOL products with an inhibition of side reactions. Among them, fine-tuning acid/base catalysts is a facile approach to tailor catalytic performance and selectivity for FAL hydrogenation to target FOL products. In addition, a bimetallic CoCu catalyst has exhibited high activity for FAL conversion under direct H_2_ gas and alcohols as the hydrogen donor due to the interaction between active Cu and Co species [35,36,46,47] and an acidity improvement in the Co–Cu system [48]. Nevertheless, the utilization of bimetallic CoCu catalysts for FOL production from FAL hydro-conversion have some drawbacks, including the low selectivity to FOL product at high conversion level, operation under high temperature, and/or the complexity of the catalyst preparation method, which are limited for an industrial production [35,36,46,47]. To tailor the basic site of the catalyst, CaO is a good candidate for investigation because CaO is a moderately basic material. It is also active and stable for FAL hydrogenation under methanol as the H_2_ source [49]. To the best of our knowledge, a combined CoCu and CaO system through a fine-tuning acid/base approach by a facile preparation method would be expected to enhance activity and selectivity for FOL production at a high conversion level under a low operating temperature.

Therefore, in this investigation, tunable Co-doping on Cu/CaO catalysts using an abundant CaO from the cement industry with different Co to Cu mole ratios were developed through a facile wetness impregnation method. Their catalytic performances were evaluated for FAL hydrogenation into FOL in comparison to monometallic Cu/CaO and Co/CaO benchmarks. Insights into catalytic activity and catalyst characterizations by various techniques were comprehensively investigated. The influences of reaction temperature, reaction time, initial H_2_ pressure, and catalyst loading were systematically performed for the Co_1.40_Cu_1_/CaO catalyst. In addition, to consider the catalyst lifetime, the reusability experiment was investigated.

## 2. Experimental

### 2.1. Catalyst Preparation

A low-cost CaO material obtained from a local cement company (TPI Polene Public Company Limited, Bangkok, Thailand) in Thailand was calcined at 900 °C for 5 h in stagnant air before utilization as support throughout this investigation. Bimetallic CoCu/CaO catalysts with different Co to Cu mole ratios was synthesized by a wetness impregnation method and compared with monometallic Cu/CaO and Co/CaO catalysts. The monometallic Cu/CaO and Co/CaO catalysts as the catalyst benchmarks were prepared by dropping a solution of copper (II) nitrate trihydrate [Cu(NO_3_)_2_·3H_2_O; CARLO ERBA, purity ≥ 99.5%] or cobalt (II) nitrate hexahydrate [Co(NO_3_)_2_·6H_2_O; CARLO ERBA, purity ≥ 98%] into deionized water on a CaO support. Similarly, bimetallic CoCu/CaO catalysts with different Co to Cu mole ratios were synthesized by dissolving a mixture of copper (II) nitrate trihydrate and cobalt (II) nitrate hexahydrate in deionized water and further dropping on a CaO support. All the resultant samples were first removed from the water at 110 °C for 12 h and subsequently calcined in stagnant air at 500 °C for 5 h. The bimetallic CoCu/CaO with Co to Cu mole ratios of 0.49:1, 0.96:1, 1.40:1, and 1.94:1 was named Co_0.49_Cu_1_/CaO, Co_0.96_Cu_1_/CaO, Co_1.40_Cu_1_/CaO, and Co_1.94_Cu_1_/CaO catalysts throughout this investigation, respectively. Moreover, the catalysts were utilized in metallic form by the reduction under a H_2_ atmosphere at 500 °C for 3 h before FAL hydrogenation experiments.

### 2.2. Catalyst Characterization

The Brunauer–Emmett–Teller (BET) surface area (SBET), pore volume (Vp), and average pore diameter (Dp) of the calcined samples were analyzed using N2 adsorption and desorption techniques (Bel Sorp mini II, Osaka, Japan). The calcined catalysts were degassed at 200 °C for 3 h before the analysis. The elemental composition in oxide form was obtained using a wavelength dispersive X-ray fluorescence spectrometer (WDXRF, Rigaku ZSX Primus, Tokyo, Japan). A chemisorption apparatus (BELCAT-B Instruments, Fukushima, Japan) was used for the hydrogen temperature-programmed reduction (H2-TPR), ammonia temperature-programmed desorption (NH3-TPD), and carbon dioxide temperature-programmed desorption (CO_2_-TPD) analyses. For the H2-TPR experiments, the calcined samples were dried in situ at 200 °C for 1 h under an inert gas, H2-TPR profiles were obtained by rising the temperature from 100 °C to 800 °C with a heating rate of 10 °C min^−1^ under a flow of 5% H2 in Ar (*v*/*v*), and detection was implemented using a thermal conductivity detector (TCD). For the NH3-TPD and CO_2_-TPD experiments, prior to implementation, the active metallic forms of calcined samples were obtained by an in situ reduction process at 500 °C for 2 h under 5% H_2_ in Ar (*v*/*v*). NH_3_ or CO_2_ adsorption was performed at 50 °C for 2 h, followed by the removal of unabsorbed NH_3_ or CO_2_ under a He gas. The profiles of NH_3_-TPD and CO_2_-TPD were recorded in the temperature range of 100 °C to 800 °C with a heating rate of 10 °C min^−1^ under an inert gas using a TCD detector. The phase identification of calcined and reduced catalysts was examined although X-ray diffraction (XRD) analysis (D8 ADVANCE, Bruker, Ltd., Bremen, Germany) using Cu Kα radiation at 40 kV and 30 mA over a 2θ range of 10–80°, at a scan speed of 2° min^−1^. The oxidation states of the reduced catalyst were investigated by X-ray photoelectron spectroscopy (XPS) using a Kratos Axis ULTRA^DLD^ (Kratos) spectrometer equipped with an Al Kα radiation source. The catalysts were reduced ex situ at 500 °C for 3 h, and further passivated under a flow of 1% O_2_ in air (*v*/*v*) at room temperature for 2 h before XPS implementations. The microstructure of the catalyst was measured using field emission scanning electron microscopy (FE-SEM) (Hitachi SU8030). Moreover, the morphology, corresponding particle size distribution, and catalyst structure observed by lattice fringes were analyzed using transmission electron microscopy (TEM) (JEOL/JEM-2100Plus) at 200 kV, accompanied by energy-dispersive X-ray spectrometry (EDS).

### 2.3. Evolution of Catalytic Performance for FAL Hydrogenation to FOL

The catalytic performance for FAL hydrogenation to FOL was evaluated in a 100 mL high-pressure batch reactor with a mixture of 1 g of FAL feedstock (Sigma–Aldrich, Bangkok, Thailand 99%), 0.1 to 0.5 g of reduced catalyst, and 40 mL of 2-propanol as an organic solvent (QREC Chemical, AR grade, >99%). The inside air was removed from the reactor by purging N_2_ at least three times and filling it with H_2_ to the target pressure. Subsequently, the reactor was heated to the desired temperature and held for different reaction times. After the reaction, the reactor’s temperature was suddenly dropped to room temperature by immersing it in an ice-cool container. The catalytic activities for Co/CaO, Cu/CaO, and CoCu/CaO with different Co to Cu mole ratios were evaluated at 120 °C, an initial H_2_ pressure of 20 bar, a reaction time of 2 h, and a catalyst loading of 20%, based on the initial mass of FAL feedstock. The effects of reaction temperature (80–140 °C), reaction time (1–10 h), initial H_2_ pressure (10–50 bar), and catalyst loading (10–50%) were further examined for the Co_1.40_Cu_1_/CaO catalyst. Moreover, duplicate or triplicate experiments with the standard deviation (SD) for each condition were reported. Lastly, the deactivation of the Co_1.40_Cu_1_/CaO catalyst was investigated at a reaction temperature of 100 °C, 20 bar H_2_ pressure, a reaction time of 4 h, while 20% catalyst loading under equilibrium conversion was not attained. The reusability of the catalyst for each consecutive run was performed by calcination at 500 °C in stagnant air for 3 h, followed by H_2_ reduction at 500 °C for 2 h.

A gas chromatography with a flame ionization detector (GC-FID, GC 2014, Shimadzu) equipped with a capillary column (DB5, 30 m × 0.32 mm × 0.1 μm) was used to identify the composition of the liquid product after catalyst separation. The injection and detection temperatures were fixed above the boiling points of FAL, FOL, and THFA at 250 °C. The oven temperature was controlled by a temperature-programmed method starting at 40 °C to 200 °C, according to our previous investigations [3,48]. The calibration curves of FAL, FOL, and THFA were performed to quantify the amount of remaining FAL and generated FOL and THFA in the liquid product after the reaction. The FAL conversion, FOL and THFA yields, and FOL selectivity were calculated according to the following Equations (1)–(3):

(1) $$\mathrm{FAL}\;\mathrm{conversion}\;\left(\%\right)=\left(\frac{\mathrm{initial}\;\mathrm{mole}\;\mathrm{of}\;\mathrm{FAL}-\mathrm{final}\;\mathrm{mole}\;\mathrm{of}\;\mathrm{FAL}}{\mathrm{initial}\;\mathrm{mole}\;\mathrm{of}\;\mathrm{FAL}}\right)\times 100$$

(2) $$\mathrm{FOL}\;\mathrm{or}\;\mathrm{THFA}\;\mathrm{yield}\;\left(\%\right)=\left(\frac{\mathrm{mole}\;\mathrm{of}\;\mathrm{FOL}\;\mathrm{or}\;\mathrm{THFA}}{\mathrm{initial}\;\mathrm{mole}\;\mathrm{of}\;\mathrm{FAL}\;}\right)\times 100$$

(3) $$\mathrm{FOL}\;\mathrm{selectivity}\;\left(\%\right)=\left(\frac{\mathrm{FOL}\;\mathrm{yield}}{\mathrm{FAL}\;\mathrm{conversion}}\right)\times 100$$

## 3. Results and Discussion

### 3.1. Fine-Tuning the Activities of Cu/CaO Catalysts by Co-Doping for FAL Hydrogenation to FOL

The catalytic transfer hydrogenation (CTH) of furfural (FAL) into furfuryl alcohol (FOL) was first examined using bare CaO and Co_1.40_Cu_1_/CaO catalysts at a reaction temperature of 120 °C, a reaction time of 2 h, and an initial N_2_ pressure at 20 bar under 2-propanol as a H_2_ donor. It was found that the catalytic activity for CTH of FAL over the bare CaO catalyst did not exhibit a FAL conversion and yield of FOL, while the Co_1.40_Cu_1_/CaO catalyst gave a FAL conversion and FOL yield of less than 1% under the investigated conditions. These results confirmed that conversion of FAL into FOL over the CoCu/CaO catalyst did not mainly undergo the CTH reaction toward the Lewis acid-mediated intermolecular hydride transfer or the Meerwein−Ponndorf−Verley (MPV).

To fine-tune the catalyst compositions, the Cu/CaO catalysts with Co doping at different Co to Cu mole ratios were synthesized, and their catalytic activities were evaluated for FAL hydrogenation to the target FOL product under a supply of direct H_2_ gas. Table 1 summarizes the FAL conversion, FOL yield, and FOL selectivity for different catalysts under similar evaluation conditions at a temperature of 120 °C, H_2_ pressure of 20 bar, and reaction time of 2 h. It should be first noted that the FOL selectivity was almost 100% for all the investigated catalysts, confirming their high selectivity to the target FOL product. It was found that monometallic Cu/CaO and Co/CaO as the benchmark catalysts exhibited FAL conversion with equal product yields of 38% and ~9.5%, respectively. Surprisingly, the FAL conversion and product yield significantly increased after introducing Co species into CaO catalysts with a fixed Cu content. When increasing the Co to Cu mole ratio from 0.49 to 1.94, the complete FOL conversion and FOL yield at >99.9% were obtained at 1.40, while a further increase up to 1.94 remained unchanged in the FAL conversion and yield of FOL. This result indicated that a Co to Cu molar ratio of 1.40:1 was sufficient for FAL conversion to the target FOL. As detected in GC chromatograms, only peaks of 2-propanol and the FOL product were detected in the liquid product using the Co_1.40_Cu_1_/CaO and Co_1.94_Cu_1_/CaO catalysts, thereby indicating their high selectivity to the FOL product with an inhibition of undesired products (see Figure S1). To examine the incorporative effects of CoCu species and CaO support, the activities of unsupported Co_1.40_Cu_1_, bare CaO, and physically mixed Co_1.40_Cu_1_ and CaO were tested under the same operating conditions. As listed in Table 1, the unsupported Co_1.40_Cu_1_ catalyst gave the FAL conversion and FOL yield at 33.5%, while the lowest conversion and FOL yield for the bare CaO support was observed. In the case of the physical mixed Co_1.40_Cu_1_ and CaO support, a lower FAL conversion and FOL yield than those of the unsupported support were detected, and these found to be 15.7%. These results confirmed that the incorporative effects of CoCu species and CaO after simultaneous calcination and reduction processes remarkably boosted the catalytic activity and selectivity for FAL hydrogenation to the target FOL product under a low operating temperature.

To compare our catalytic performance with the findings of previous investigations, we summarized the recent development of non-noble catalysts for FAL hydrogenation to FOL in a batch reactor under an H_2_ atmosphere (see Table 2). It was found that the activity and selectivity of our developed Co_1.40_Cu_1_/CaO catalyst were comparable to other recent reports at lower or moderate operating temperatures. As summarized in Table 2, the NiCoZn@CN-600 and Ni_0.5_@OMC-600 catalysts gave the conversion and yield reaching nearly 100% at temperatures of 160 °C and 180 °C, respectively. Lower operating temperatures (110–120 °C) with high conversion and yield of FOL were obtained using PMS-2-CuCo, Na−Cu@TS-1, and ZnCo-US@NC-700 catalysts synthesized by sol-gel hydrothermal, combined isothermal and ion exchange, and ultrasonic-assisted methods, respectively. Nevertheless, operation under severe conditions and the complexity of catalyst preparation methods are not preferable for industrial applications. Therefore, based on our findings, the correlation between activities for FAL hydrogenation and catalyst properties would be interesting for the first-time investigation in bimetallic Co-Cu and CaO systems and, as such, will be provided in the next sections.

### 3.2. Physical and Chemical Properties of the Catalysts

#### 3.2.1. Elemental Composition and Textural Properties of the Catalysts

The composition of calcined samples was first evaluated using XRF analysis. The oxygen content was calculated based on subtraction from the other components detected by XRF measurements. As summarized in Table 3, Ca was the major component of the support material obtained from the local cement company in Thailand. Furthermore, the actual metal loadings of all the catalysts were nearly comparable to the calculation of metal nitrate sources.

The textural properties of the as-synthesized catalysts in oxide form were evaluated using N_2_ sorption measurements. The isotherms of N_2_ adsorption and desorption and catalyst pore size distribution using the BJH approach are depicted in Figure S2. The type of isotherm for bare CaO calcined at 900 °C exhibits a type III isotherm, indicating a macroporous structure due to the weak adsorption ability of the support interaction with N_2_ molecules. This was in line with previous investigation [50]. Also, type III isotherms with characteristics of macroporous material [51] or interparticle features were observed after the addition of metals into the support, with clear detection for Co_0.96_Cu_1_/CaO and Co_1.40_Cu_1_/CaO, and Co_1.94_Cu_1_/CaO samples, according to the IUPAC classification (Figure S2A). It should be noted that adding Co and Cu species into the support exhibited a self-tunable textural structure with a reduction in pore diameter of the catalysts, which might be suitable for FAL hydrogenation. In addition, the pore size distribution calculated using the Barrett–Joyner–Halenda (BJH) method revealed a more uniform distribution after incorporation between the metals and support (Figure S2B).

Table 3 lists the specific surface area (S_BET_), pore volume (V_p_), and pore diameter (D_p_) of the support and he as-calcined catalysts. The specific surface area of the bare support was 7 m^2^ g^–1^, with a pore volume of 0.097 cm^3^ g^–1^. The addition of monometallic Cu and Co catalysts to the support resulted in a decrease in catalyst surface area, pore volume, and pore diameter, thereby indicating the blockage or coverage of pores by Cu or Co species. Interestingly, in the case of bimetallic CoCu/CaO catalysts, an increase in specific surface area and pore volume, accompanied by a reduction in pore diameter, was observed when the mole ratio of Co to Cu increased from 0.49 to 1.40. This result suggested that the introduction of Co into Cu species resulted in the formation of a larger catalyst surface area and pore volume with a smaller pore diameter. However, the further addition of Co into Cu up to 1.94 did not improve the textural properties of the catalyst, thereby suggesting an agglomeration of metal species. It should be noted that the Co_1.40_Cu_1_/CaO catalyst exhibited better physical properties than the other supported catalysts. 

#### 3.2.2. Reducibility of the Catalysts

**Figure 1:**
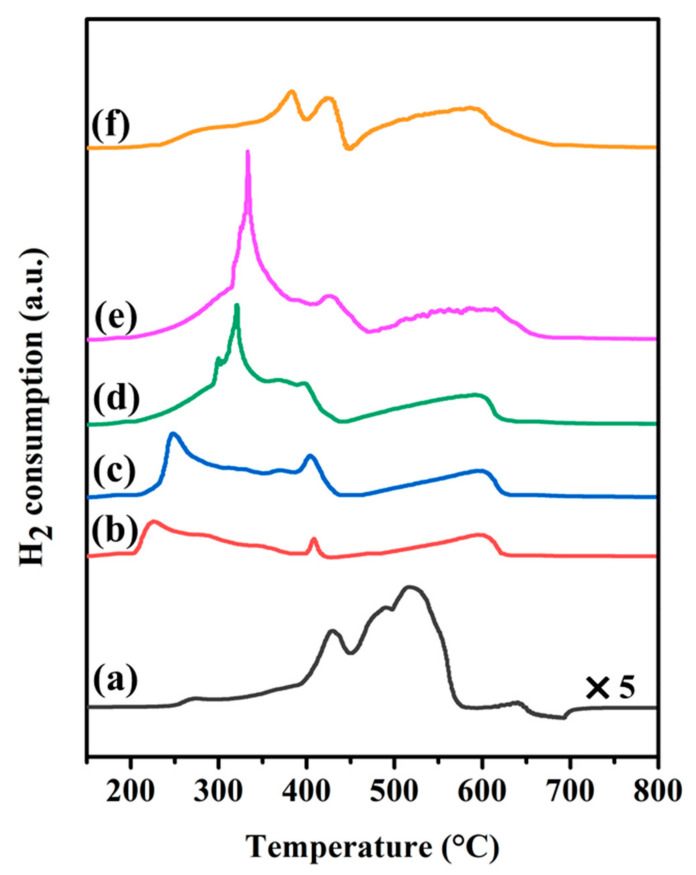
H_2_-temperature programmed reduction (H_2_-TPR) profiles of calcined (a) Cu/CaO, (b) Co_0.49_Cu_1_/CaO, (c) Co_0.96_Cu_1_/CaO, (d) Co_1.40_Cu_1_/CaO, (e) Co_1.94_Cu_1_/CaO, and (f) Co/CaO samples.

The reduction behavior and metal-support interactions of the as-calcined samples were investigated using temperature-programmed reduction (TPR) under the dilution of H_2_ gas (see Figure 1). As exhibited in Figure S3, H_2_-TPR experiments of the bulk Cu, Co, and CoCu species, prepared by calcination of metal nitrate precursors at 500 °C, were initially conducted to elucidate the natural reduction of unsupported samples. The broad reduction peaks at 342 °C are assigned to the simultaneous reduction of CuO to metallic Cu, while the reduction of bulk Co is detected in the wide range of 260 °C to 495 °C, with a maximum at 395 °C, which indicates the transformation of the Co_3_O_4_ spinel-type structure into metallic Co. For bulk CoCu species, the reduction peak shifts to a lower temperature (≈280 °C) than that of bulk Cu and Co species, thereby confirming that the addition of Co species into Cu species facilitated the reduction of bimetallic CoCu species. This result was associated with the hydrogen spillover effect from Cu species into neighboring Co species [52,53,54].

For supported samples, Figure 1 reveals the H_2_-TPR profiles of Cu/CaO, Co_0.49_Cu_1_/CaO, Co_0.96_Cu_1_/CaO, Co_1.40_Cu_1_/CaO, Co_1.94_Cu_1_/CaO, and Co/CaO samples. The reduction profile of the Cu sample exhibits two major irregular reduction peaks, accompanied by a shift to higher temperatures compared with bulk Cu, implying the behavior of strong metal-support interactions. The former reduction peaks with smaller peak areas at 435 °C are due to the reduction of smaller Cu particles on the catalyst surface, whereas the latter accumulated broad peaks in the wide range of 450 to 570 °C are assigned to the bulk Cu species strongly interacting with the CaO support material. For the monometallic Co/CaO sample, it is generally known that the reduction of Co oxide species occurs in two steps (Co_3_O_4_ to CoO and CoO to metallic Co) [55]. Consistently, the reduction peak at 380 °C is due to the reduction of Co^3+^ to Co^2+^, while the reduction peak at 425 °C is due to the reduction of Co^2+^ to metallic Co. On the other hand, the broad reduction peak in the wide range of 450 to 680 °C is assigned to the reducibility of stronger Co species interacting with the CaO support. In the case of bimetallic CoCu-supported samples, it is apparent that the reduction temperature shifts to a lower temperature than that of supported Cu and Co samples, thereby indicating that the incorporation of Co and Cu species facilitated the reducibility of the bimetallic system. As detected in the H_2_-TPR profiles of the Co_0.49_Cu_1_/CaO and Co_0.96_Cu_1_/CaO samples, three similar reduction peaks are observed, thereby indicating that several reduction steps occurred to complete the transformation of metal oxide to metallic form. The increase in reduction peak at 235 °C for Co_0.49_Cu_1_/CaO and 250 °C for Co_0.96_Cu_1_/CaO following the introduction of Co loading is attributed to the simultaneous reduction of smaller CoCu particles. This result was consistent with previous observations for the CoCu/Al_2_O_3_ catalyst [48]. Otherwise, the second reduction peak at 405 °C for both samples may be due to the reduction of bulk or larger CoCu particles. Similarly, a broad reduction peak in the wide range of 450 to 625 °C is due to the reduction of stronger interactions between metals and the support. In the case of higher Co loading for the Co_1.40_Cu_1_/CaO and Co_1.94_Cu_1_/CaO samples, the reduction patterns are similar to those of lower Co loading for the Co_0.49_Cu_1_/CaO and Co_0.96_Cu_1_/CaO samples. However, the peak intensity at ≈340 °C significantly increases with Co loading, accompanied by a shift to a higher reduction temperature. This may be due to the formation of larger CoCu particles, resulting in difficulty of reduction for bimetallic CoCu samples. In contrast, the increase in peak intensity is due to the large amount of hydrogen needed to generate the metallic species. Therefore, a moderate reduction temperature was selected at 500 °C throughout the investigations for transformation of most metal oxide species to metallic forms, as confirmed by XRD and XPS experiments.

#### 3.2.3. Structural Properties of the Catalysts

**Figure 2:**
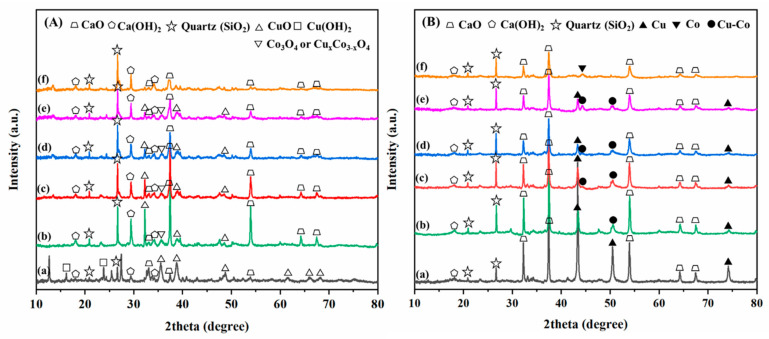
X-ray diffraction patterns of (**A**) calcined and (**B**) reduced (a) Cu/CaO, (b) Co_0.49_Cu_1_/CaO, (c) Co_0.96_Cu_1_/CaO, (d) Co_1.40_Cu_1_/CaO, (e) Co_1.94_Cu_1_/CaO, and (f) Co/CaO catalysts.

The structural properties of the catalysts were examined using XRD and XPS measurements. Figure 2 displays the XRD patterns of calcined and reduced Cu/CaO, Co_0.49_Cu_1_/CaO, Co_0.96_Cu_1_/CaO, Co_1.40_Cu_1_/CaO, Co_1.94_Cu_1_/CaO, and Co/CaO samples. As observed in the calcined catalysts (see Figure 2A), the appearance of Ca used as a support material in our investigations was in the major form of CaO and minor forms of Ca(OH)_2_ and quartz (SiO_2_) (JCPDS No. 146-1045) [56]. The detection of Ca(OH)_2_ was attributable to the reaction between CaO and environmental moisture due to its highly hygroscopic nature [57,58,59]. For the calcined monometallic Cu/CaO catalyst, the diffraction peaks at 2θ values of 35.3°, 38.8°, and 61.8°, corresponding to planes of (002), (111), and (-113), respectively, are assigned to the phase of CuO (JCPDS No. 80–1916) [60], respectively, whereas the phase of Cu(OH)_2_, a reaction between CuO and H_2_O, is detected at 2θ values of 16.3° and 24° in planes of (020) and (021) (JCPDS No. 13−420) [61], respectively. Additionally, the unknown diffraction peaks in a 2θ range of 10° to 27° might be attributed to the phase of interacted Ca and Cu oxide species, which disappears after reduction at 500 °C under a flow of H_2_ (see Figure 2B). For the calcined monometallic Co/CaO catalyst, the diffraction peaks assigned to the Co_3_O_4_ phase were not detectable, implying that small Co particles with high dispersion on the support were formed after calcination. For calcined bimetallic CoCu/CaO catalysts with different Co to Cu mole ratios, in part due to the detection of CuO, the diffraction peaks at a 2θ of 36° detected for all the bimetallic catalysts are attributed to the spinel Co_3_O_4_ or Cu_x_Co_3−x_O_4_ phase [62,63,64].

In the case of reduced catalysts, as shown in Figure 2B, it was found that the diffraction peaks detected for all the samples at 2θ values of 32.3°, 37.2°, 54.7°, 64.6°, and 67.4°, attributed to planes of (111), (200), (202), (311), and (222), respectively (JCPDS No. 82-1691) [65], were assigned to the CaO phase, thereby indicating the dominant characteristic feature of the support material after reduction. In addition, the minor phase of Ca(OH_2_) (JCPDS No. 44-1481) [66] along with quartz (SiO_2_) (JCPDS No. 146-1045) [56] correspondingly detected for all the samples would be impurities of the support material of our investigation. In the case of the reduced monometallic Cu/CaO catalyst, the phase of metallic Cu is observed at 2θ values of 43.7°, 51.0°, and 74.3°, corresponding to planes of (111), (200), and (220), respectively (PDF No. 00-04-0836) [3,54]. For the reduced monometallic Co/CaO, the peak at a 2θ of 44.5° is ascribed to metallic Co in the (111) plane (JCPDS file No. 15-0806) [67]. Otherwise, in the case of bimetallic CoCu/CaO catalysts with different Co to Cu mole ratios, diffraction peaks at 2θ values of 43.7° and 74.3° assigned to metallic Cu were detected, whereas diffraction peaks at 2θ values of 44.2° and 51.2° seemed to be the formation of a CuCo alloy in the (111) and (200) planes [68], respectively. This result was in agreement with a previous investigation showing that the formation of a CoCu alloy could be generated after reduction at temperatures higher than 400 °C under a H_2_ atmosphere [48,63,64,69,70]. Additionally, the formation of an alloy would be confirmed by HRTEM analysis into the next section. In addition, the peak intensity at a 2θ of 51.2° meaningfully decreases with increasing Co loading, implying that a smaller crystallite size was obtained after the addition of Co species into Cu species. Therefore, based on XRD analysis, the developed CoCu/CaO catalysts coexisted in the form of metallic Cu and CoCu alloys. Furthermore, the synergetic properties between the Cu and Co species to form the bimetallic CoCu phase would be beneficial for FAL hydrogenation [36].

**Figure 3:**
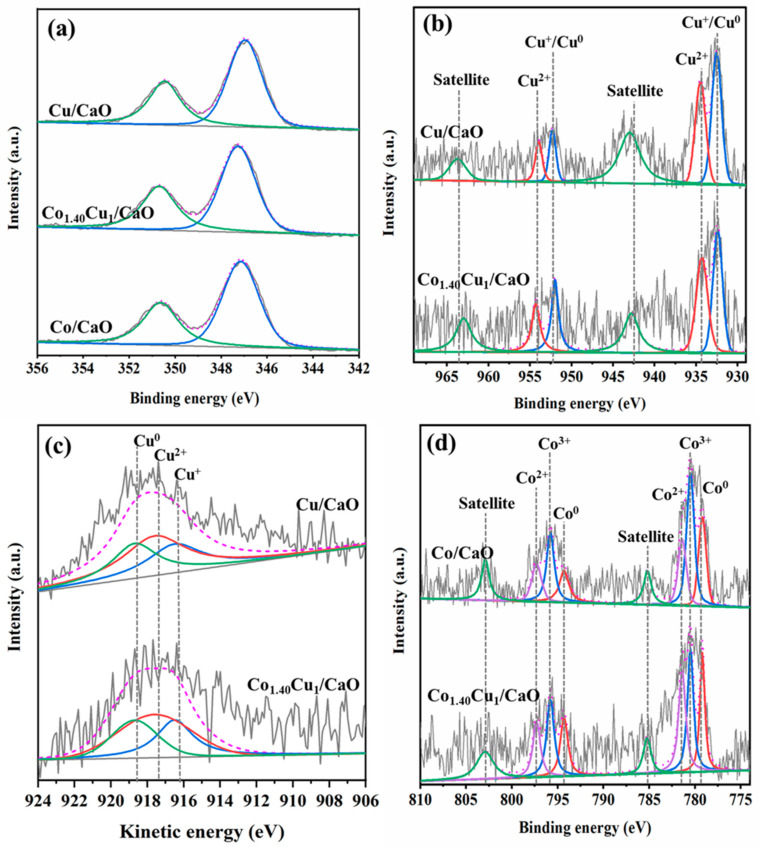
XPS spectra of (**a**) Ca*2p* of reduced Cu/CaO, Co_1.40_Cu_1_/CaO, and Co/CaO catalysts, (**b**) Cu*2p* of reduced Cu/CaO and Co_1.40_Cu_1_/CaO catalysts, (**c**) Cu LMM of reduced Cu/CaO and Co_1.40_Cu_1_/CaO catalysts, and (**d**) Co*2p* of reduced Co/CaO and Co_1.40_Cu_1_/CaO catalysts.

The oxidation states of active metal species for the reduced Cu/CaO, Co/CaO, and Co_1.40_Cu_1_/CaO catalysts were further elucidated using XPS analysis (Figure 3). The catalysts were reduced at 500 °C for 3 h under a flow of H_2_ gas accompanied by catalyst passivation using 1% O_2_ in an inert gas at room temperature before XPS implementation. Table S1 summarizes the binding energies and kinetic energies of different species from the XPS spectra. As shown in Figure 3a, the XPS spectra of Ca*2p* exhibit two major binding energies at 350.8 eV and 347 eV with a peak separation of ~3.5 for all the catalysts, which are characteristic of Ca^2+^ [71,72]. In the case of active metal species, the XPS spectra of Cu*2p,* CuLMM, and Co*2p* are revealed in Figure 3b–d. The Cu*2p* spectra detected for the reduced Cu/CaO, and Co_1.40_Cu_1_/CaO catalysts confirmed two distinct doublet peaks of Cu*2p*_1/2_ and Cu*2p*_3/2_ (see Figure 3b). Shake-up satellite peaks are detected for both Cu-containing catalysts, indicating the appearance of Cu^2+^ species. Taking into account the deconvoluted peaks, the peaks at binding energies of 934.5 eV and 954.3 eV belong to Cu^2+^, whereas the existence of Cu^1+^/Cu^0^ species exhibits peaks at binding energies of 932.4 eV and 952.0 eV [48], confirming the remaining Cu^2+^ on the catalyst surface for both catalysts. To further identify Cu^2+^, Cu^1+^, and Cu^0^ species, the spectra of the Cu LMM auger, as revealed in Figure 3c, indicated three different peaks located at kinetic energies of approximately 918.7 eV, 917.6 eV, and 916.5 eV, corresponding to Cu^0^, Cu^2+^, and Cu^1+^ states [73,74], respectively, thereby indicating the combination of triple-valence states after reduction at 500 °C. Furthermore, the remaining Cu^0^, Cu^1+^_,_ and Cu^2+^ states on the catalyst surface would be advantageous for FAL hydrogenation. Adsorption and dissociation of hydrogen could preferably occur by metallic Cu species, while carbonyl groups (C=O) outside the furan ring could interact by oxophilic Cu^1+^ and Cu^2+^ states, resulting in the facilitated FAL hydrogenation step to target the FOL product [3,52]. In the case of Co-containing catalysts, as demonstrated in Figure 3d, the Co*2p* spectra of reduced Co/CaO and Co_1.40_Cu_1_/CaO catalysts exhibits two doublet peaks for metallic Co (Co^0^) and Co oxides, with corresponding shake-up satellite peaks (Co^2+^ and Co^3+^). The peaks at binding energies of 779.2 eV and 794.3 eV attributed to Co*2p*_3/2_ and Co*2p*_1/2_, respectively, are ascribed to metallic Co [47,75]. Additionally, the existence of Co^3+^ exhibits peaks at binding energies of 780.5 eV and 795.8 eV, whereas peaks at binding energies of 781.4 eV and 797.3 eV are assigned to Co^2+^. These results confirmed the coexistence between metallic Co and Co oxides. Based on H_2_-TPR and XRD analyses, although Cu and Co oxides were not noticed in the XRD patterns of the reduced Cu/CaO, Co/CaO, and Co_1.40_Cu_1_/CaO catalysts (see Figure 2B), these occurrences might be due to oxidation on the catalyst surface by air exposure before the XPS experiments and/or an incomplete reduction at 500 °C observed in the H_2_-TPR profiles.

#### 3.2.4. Acidity and Basicity of the Catalysts

**Figure 4:**
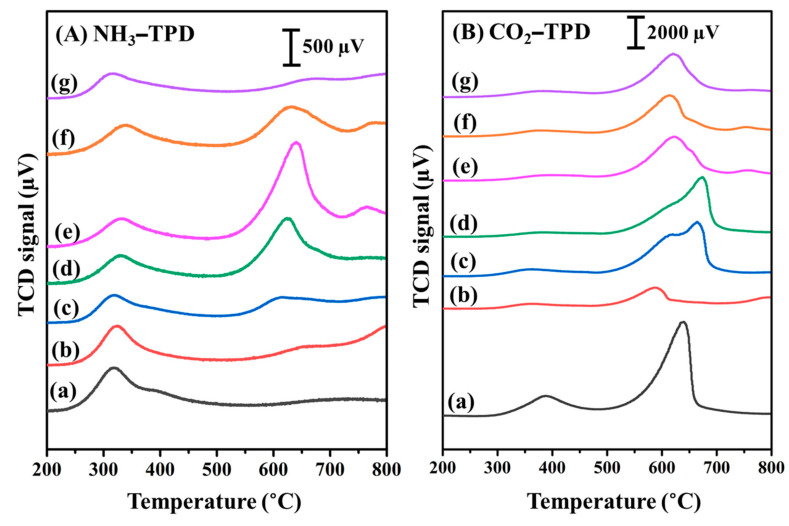
(**A**) NH_3_ and (**B**) CO_2_ temperature-programmed desorption profiles of reduced (a) CaO support, (b) Cu/CaO, (c) Co_0.49_Cu_1_/CaO, (d) Co_0.96_Cu_1_/CaO, (e) Co_1.40_Cu_1_/CaO, (f) Co_1.94_Cu_1_/CaO, and (g) Co/CaO catalysts.

The number of acid/base sites generated from active metal species and the CaO support plays a significant role in tuning the activity and selectivity of the catalyst. Temperature-programmed desorption using NH_3_ and CO_2_ as probe molecules for acidity and basicity, respectively, was investigated for all the reduced catalysts (Figure 4). As observed in the NH_3_-TPD profiles, as demonstrated in Figure 4A, all the reduced catalysts exhibited a desorption peak in the range of 200–450 °C, representing the availability of acid sites of the reduced catalysts at low temperature. Moreover, after Cu, CoCu, and Co addition, the peak intensity at a low temperature slightly decreased compared to that of the bare CaO support. Nevertheless, considering the range of temperatures higher than 500 °C, the introduction of Co species into Cu species for the Co_0.96_Cu_1_/CaO and Co_1.40_Cu_1_/CaO catalysts resulted in a dramatic increase in the stronger acid sites in the temperature range of 500–750 °C, implying that the cooperative interaction between the CoCu species and CaO support improved the sites of acid. Unexpectedly, the increase in the Co to Cu mole ratio up to 1.94 did not relatively increase the number of acidic sites. It should be noted that a high amount of Co species had a negative effect on the reducing acidic sites, as confirmed in the NH_3_-TPD profile of the monometallic Co/CaO catalyst. According to intensity of NH_3_-TPD profiles, the strong acidic sites were highest for the Co_1.40_Cu_1_/CaO catalyst, while the weak acidic sites were not meaningfully different for all the supported catalysts.

The CO_2_-TPD profiles for the CaO support and Cu/CaO, CoCu/CaO, and Co/CaO catalysts are depicted in Figure 4B. The peak intensity in the temperature range of 300–450 °C for weak basic sites and 500–710 °C for strong basic sites was highest for the CaO support among the other supported catalysts, due to the characteristic nature of the CaO-based material. These results were in line with previous observations [71,72]. In the case of the monometallic Cu/CaO catalyst, the peak intensity for both regions dropped significantly, indicating that the metallic Cu species could not tailor the basicity of the catalyst. Moreover, for the Co_0.49_Cu_1_/CaO and Co_0.96_Cu_1_/CaO catalysts, the peak intensity detected in the temperature range of 500–710 °C for strong basic sites increased, accompanied by a peak shift to a higher temperature compared with the CO_2_-TPD profile of the monometallic Cu/CaO catalyst, thereby indicating the generation of strongly interacting CO_2_ with the catalysts. Interestingly, for the Co_1.40_Cu_1_/CaO and Co_1.94_Cu_1_/CaO catalysts, the desorption peak shifts to a lower temperature, while the peak intensity decreases, similar to the CO_2_-TPD profile of the monometallic Co/CaO catalyst, which suggests that the available Co species with higher metal loading drops in the number of basic sites. Based on the comparable peak intensity of CO_2_-TPD profiles, it was found that the basicity was highest for the CaO support, followed by all the bimetallic CoCu catalysts with different basicity levels. Otherwise, the lowest basicity was observed for the monometallic Cu catalyst.

It was reported that the acidic sites of the catalyst significantly affect the hydrogenation ability of FAL to the FOL product; however, a stronger and larger number of acidic sites is not favored for selective FOL products because of the generation of undesired products, such as furfuryl isopropyl ether and furfuryl diisopropyl acetal [31]. This behavior was confirmed in previous investigations in which supported Al_2_O_3_ catalysts favored the generation of alkyl furfuryl ethers through esterification under alcohols as an organic solvent due to their extra availability of acidic sites [3,9]. Certainly, the tunable basic site by support and/or active metal species can reduce the number of acidic sites occupied on the catalyst surface to inhibit the side reactions and improve the selectivity to the FOL product [31]. Surprisingly, in our developed CoCu/CaO catalyst, the addition of active Co species to Cu species supported on the CaO catalyst can fine-tune the acid/base properties of the synthesized catalysts by a facile wetness impregnation method. As summarized in Table 1, the Co_1.40_Cu_1_/CaO and Co_1.94_Cu_1_/CaO catalysts exhibited the highest activity and selectivity for FAL hydrogenation to FOL among the other prepared catalysts. Considering acidity detected from the peak intensity, the number of acid sites in the temperature range of 500–750 °C for strong acid sites was highest for the Co_1.40_Cu_1_ catalyst, and meaningfully declined for the Co_1.94_Cu_1_ catalyst (see Figure 4A). This observation could be explained by the fact that the acidic sites facilitated FAL hydrogenation, as confirmed by the high desired FOL yield obtained from the Co_1.40_Cu_1_/CaO and Co_1.94_Cu_1_/CaO catalysts. Nevertheless, the decline in acidic sites for higher Co loading of the Co_1.94_Cu_1_/CaO catalyst did not affect the catalytic performance, implying that the available active sites from Co addition are accomplished for the reaction under the investigated conditions. Additionally, it should be noted that the Co species can tune the basic sites of the reduced catalysts to tailor the basicity of the catalyst (see Figure 4B). It was found that the basicity based on the observation of peak intensity of CO_2_ profiles in the temperature range of 500–710 °C slightly dropped when the Co to Cu mole ratio increased from 0.49 to 1.94, while the FAL conversion and FOL yield relatively increased (see Table 1). This result confirmed that adjustment of basic sites by Co addition improved catalytic activity and selectivity for FOL production. In addition, it should be further noted that the low catalytic performance for monometallic Co and Cu catalysts was due to the decrease in acid/base sites of the catalysts detected in NH_3_ and CO_2_-TPD profiles. 

#### 3.2.5. Morphology of the Catalysts

The microstructure of the CaO support and reduced Cu/CaO, Co/CaO, and Co_1.40_Cu_1_/CaO catalysts was investigated using FE-SEM (see Figure S4). As shown in Figure S4a, the microstructure of the bare CaO support was found to be a loose structure which consisted of large particles connecting to one other. After Cu and Co additions, as revealed in Figure S4b,c, the modification of the catalyst surface to a denser structure occurred, especially for the monometallic Cu catalyst. Additionally, the morphology of the reduced Co_1.40_Cu_1_/CaO catalyst was composed of irregular shapes with smaller particle sizes compared with the bare CaO support (see Figure S4d). These results were comparable to the drop in BET surface area after the addition of Cu and Co into the CaO support, in addition to the increase in BET surface area for the bimetallic Co_1.40_Cu_1_/CaO sample (see Table 3).

**Figure 5:**
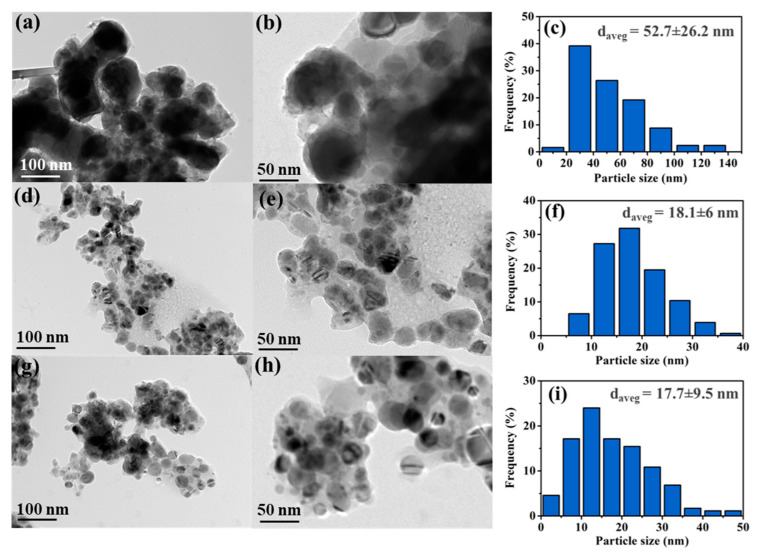
Typical TEM images and particle size distribution of reduced (**a**–**c**) Cu/CaO, (**d**–**f**) Co/CaO, (**g**–**i**) Co_1.40_Cu_1_/CaO catalysts with fixed metal loading of ~25%.

To examine the morphology, corresponding particle size distribution, and catalyst structure observed by lattice fringes, the different magnifications of TEM images and particle size distribution for the reduced Cu/CaO, Co/CaO, and Co_1.40_Cu_1_/CaO catalysts at fixed metal loading ~25% are displayed in Figure 5, and the TEM image of bare CaO support is revealed in Figure S5. It was found that all the catalysts exhibited different particle sizes, and some particles seemed to agglomerate with other particles. This might be because the CaO support has a low surface area, as confirmed by the N_2_ sorption experiment (see Table 3), resulting in agglomerated metal species outside the pores of the support. For the monometallic Cu/CaO catalyst (Figure 5a–c), uneven particles were observed, with a non-uniform particle size distribution of approximately 52.7 ± 26.2 nm. Additionally, in the case of the monometallic Co/CaO catalyst, as demonstrated in Figure 5d–f, a more uniform particle size distribution with a spherical shape and average particle size of 18.1 ± 6 nm could be obtained. Interestingly, as shown in Figure 5g–i, the particle size and shape of the bimetallic Co_1.40_Cu_1_/CaO catalyst were similar to those of the monometallic Co/CaO catalyst, indicating that Co addition interacted well with Cu species to form a smaller bimetallic structure. This result was consistent with the XRD analysis, in which a smaller crystallite size was formed after the addition of Co species in Cu species in metallic form.

**Figure 6:**
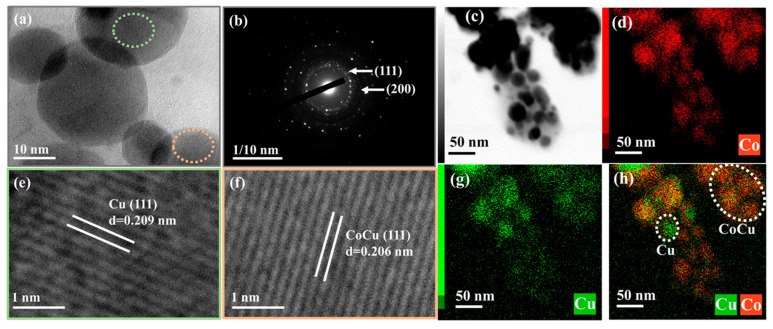
(**a**,**e**,**f**) Representative HRTEM images with (**b**) diffraction patterns and (**c**,**d**,**g**,**h**) EDS mapping of the reduced Co_1.40_Cu_1_/CaO catalyst.

The lattice fringes and dispersion of active metal species were monitored to confirm the structure of the catalyst. The HRTEM images with diffraction patterns and EDS mapping of the reduced Co_1.40_Cu_1_/CaO catalyst are revealed in Figure 6. The typical HRTEM image (Figure 6a) with clear lattice fringes, as exhibited in Figure 6e,f, confirmed that *d*-spacings of 0.206 nm and 0.209 nm were attributed to the CoCu alloy and metallic Cu, respectively, with a correspondence to the (111) plane (see Figure 6b). It was reported that the *d*-spacing between 0.205 nm for metallic Co in the plane of (111) and 0.209 nm for metallic Cu in the plane of (111) corresponded to a CoCu alloy [63,64]. Moreover, as shown in EDS mapping (Figure 6c,d,g,h), the active Co and Cu species were highly dispersed on each other’s species, while some immerged Co and Cu particles were detected in an overlay image owing to the formation of CoCu alloy (Figure 6h). However, individual Cu particles were also found, which indicated the incomplete generation of metal alloy species. Correspondingly, the coexistence of Cu and CoCu alloy was in agreement with the diffraction peaks detected in XRD analysis of the reduced Co_1.40_Cu_1_/CaO catalyst (see Figure 2B). According to the XRD and TEM measurements, smaller catalyst particle sizes and CoCu alloy formation were obtained after adding Co species into Cu species supported on the CaO catalyst.

### 3.3. Optimization of FAL Hydrogenation to FOL over the Co_1.40_Cu_1_/CaO Catalyst

**Figure 7:**
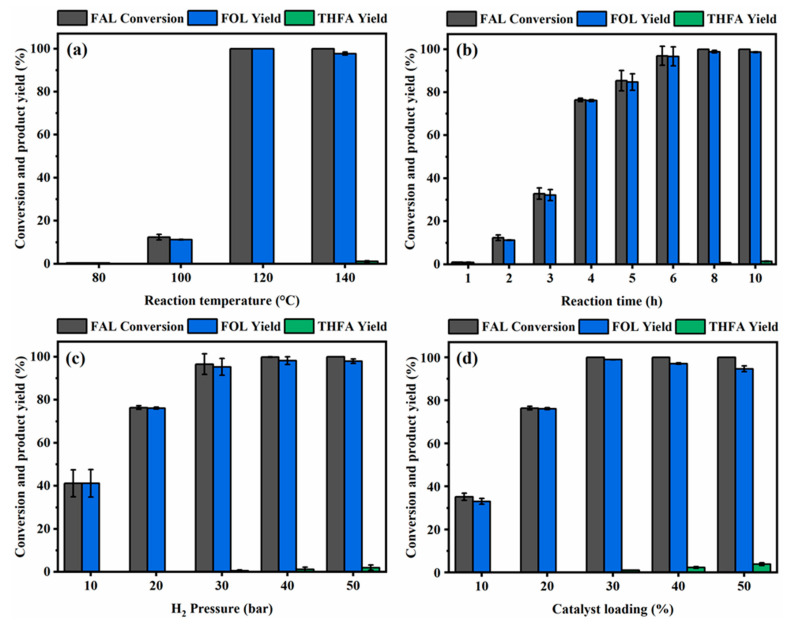
Influences of reaction temperature, reaction time, H_2_ pressure, and catalyst loading over Co_1.40_Cu_1_/CaO catalyst: (**a**) reaction conditions: 20% of catalyst loading, 20 bar of H_2_ pressure, and time of 2 h; (**b**) reaction conditions: 20% of catalyst loading, 20 bar of H_2_ pressure, and 100 °C of temperature; (**c**) reaction conditions: 20% of catalyst loading, 100 °C of temperature, and time of 4 h; and (**d**) reaction conditions: 100 °C of temperature, 20 bar of H_2_ pressure, and time of 4 h. All the experiments were conducted using 1 g of FAL feedstock in 40 mL of 2-propanol as an organic solvent.

The influences of reaction temperature, reaction time, initial H_2_ pressure, and catalyst loading were further optimized for the Co_1.40_Cu_1_/CaO catalyst, since a Co to Cu molar ratio of 1.40:1 was sufficient for FAL conversion to the target FOL (see Figure 7). The influence of reaction temperature on FAL conversion and yield of FOL under fixed H_2_ pressure at 20 bar and a time of 2 h was first investigated (Figure 7a). It was found that the FAL conversion and FOL yield were less than 1% at 80 °C, while further increasing the temperature to 120 °C resulted in a significant improvement in the catalytic performance to obtain 100% conversion and FOL yield. When the reaction temperature increased to 140 °C, the FAL conversion remained unchanged at 100%. Nevertheless, the yield of FOL dropped to 97.7%, accompanied by the generation of tetrahydrofurfuryl alcohol (THFA), thereby suggesting the occurrence of ring hydrogenation at high temperatures. As a result of the temperature effects, the reaction time was further examined at 100 °C and H_2_ pressure at 20 bar, as the lower temperature with extension time could improve the activity of the catalyst (Figure 7b). As expected, when the reaction time was extended to 6 h, almost complete FAL conversion with a 96.7% yield of FOL was achieved. Likewise, an insignificant change in FAL conversion and FOL yield was obtained after increasing the reaction time from 6 h to 10 h. To further reduce the reaction time at 100 °C with a fixed reaction time of 4 h, the effect of the initial H_2_ pressure was varied in the range of 10 to 50 bar of initial H_2_ pressure (Figure 7c). The FAL conversion and yield of FOL increased from 41.2% to 96.6% and 41.2% to 95.3%, respectively, while the H_2_ pressure increased from 10 bar to 30 bar. This could be explained by the fact that the increase in H_2_ pressure facilitated the ability of H_2_ to dissolve in the solution, followed by a reaction with FAL at active sites on the catalyst surface. This explanation was in line with a previous investigation [76].

Furthermore, the generation of the undesired THFA product attained the highest value at 2% THFA yield under 50 bar H_2_ pressure, thereby confirming that the activity for ring hydrogenation of FAL to THFA was promoted by H_2_ pressure. Additionally, the effect of catalyst loading varied from 10% to 50% by the weight of FAL feedstock at a temperature of 100 °C, 20 bar H_2_ pressure, and a reaction time of 4 h (Figure 7d). The FAL conversion and FOL yield reached 100% and 98.9%, respectively, at a catalyst loading of 30%. Finally, further adding a catalyst amount up to 50% loading resulted in an undesired THFA yield of 3.9% due to the hydrogenation of C=C in the furan ring. It can be concluded that FAL hydrogenation to the FOL product over fine-tuned CoCu supported on the CaO catalyst could certainly inhibit the generation of undesired products by side reaction suppression.

The high performance of CoCu and CaO system could be explained based on the results of catalyst characterization and catalytic activity. Additionally, the reaction mechanism for FAL to FOL product was discussed based on the previous reports. It was first noted that the Co addition to Cu on the CaO support could fine-tune the appropriate acidic and basic sites (NH_3_-TPD and CO_2_-TPD experiments). Meanwhile, smaller catalyst particle sizes and the formation of CoCu alloy were obtained after adding Co species into Cu species supported on the CaO catalyst, and further reduction at 500 °C in a flow of H_2_ (XRD and TEM observations). The combined XRD and XPS analysis confirmed the coexistence between Cu and Co species in the form of metal and metal oxides after catalyst reduction. The remaining metal and metal oxides on the catalyst surface would be advantageous for FAL hydrogenation. This could be explained that the adsorption and dissociation of hydrogen could preferably occur by metallic Cu and Co species, while carbonyl groups (C=O) outside the furan ring could interact by oxophilic Cu^1+^, Cu^2+^, Co^2+^, and Co^3+^ states, resulting in the facilitation of FAL hydrogenation step to target the FOL product [3,36,41,52]. These comprehensive investigations could elucidate that the high activity and selectivity are mainly attributed to the synergistic effect between the active CoCu species on CaO with fine-tune the proper acidic and basic sites.

### 3.4. Reusability Experiment of the Co_1.40_Cu_1_/CaO Catalyst

**Figure 8:**
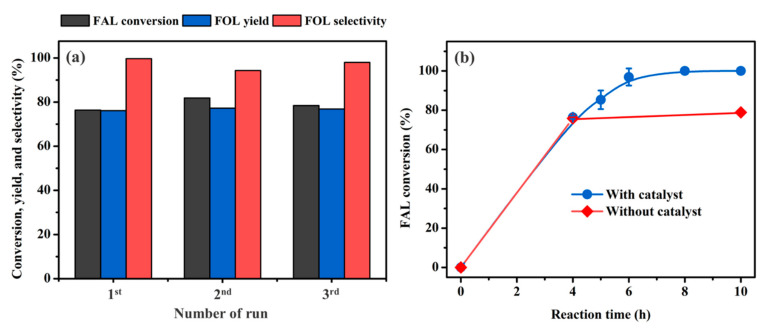
(**a**) Reusability of the Co_1.40_Cu_1_/CaO catalyst at a reaction temperature of 100 °C, 20 bar H_2_ pressure, and a reaction time of 4 h and (**b**) metal leaching investigation at a reaction temperature of 100 °C and 20 bar H_2_ pressure.

The reusability of the optimal Co_1.40_Cu_1_/CaO catalyst was investigated by operating the reaction at a temperature of 100 °C, H_2_ pressure of 20 bar, a reaction time of 4 h, and a catalyst loading of 20% (see Figure 8). After each experiment, the spent catalyst was filtered, before being washed with ethanol several times. As reported previously, the catalysts would be recommended to be reduced under H_2_ flow after each consecutive run [21,31,77]. Therefore, after each consecutive experiment, the catalyst was calcined at 500 °C in stagnant air combined with reduction at 500 °C in a flow of H_2_. As shown in Figure 8a, the FAL conversion and FOL yield and selectivity remained constant throughout the three consecutive runs. These results confirmed that the developed Co_1.40_Cu_1_/CaO catalyst was highly stable under these investigated conditions. For extended investigation, as shown in Figure 8b, the catalyst in the liquid product after 4 h of reaction time was removed, and the remaining liquid product was further tested for another 6 h at a reaction temperature of 100 °C and 20 bar H_2_ pressure. It was found that the conversion was nearly constant in the time period from 4 h to 10 h, confirming that metal leaching could be eliminated for an implementation of our developed catalyst. 

## 4. Conclusions

We successfully fine-tuned the CoCu and CaO systems with different Co to Cu mole ratios via a simple wetness impregnation method, and their catalytic activities were evaluated for the hydrogenation of furfural (FAL) into furfuryl alcohol (FOL). The optimal Co_1.40_Cu_1_/CaO catalyst demonstrated excellent catalytic performance with 100% conversion and 98.9% yield of FOL (100 °C, 20 bar H_2_, and 4 h) in comparison to Cu/CaO and Co/CaO benchmarks. The Co addition to Cu could result in a simple reduction behavior of the CoCu system. The formation of major Cu and CoCu alloys was observed after reduction, which played crucial roles in the reaction. Surprisingly, an enhanced FAL hydrogenation to a highly selective FOL product with the inhibition of undesired products was due to optimal acid and base properties and better catalyst textures with smaller CoCu sizes. Moreover, the activities of the Co_1.40_Cu_1_/CaO catalyst remained unchanged throughout the third consecutive experiments. This investigation provided a novel CoCu and CaO system for boosting the yield and selectivity of the target FOL product toward FAL hydrogenation.

## Figures and Tables

**Table 1:** Catalytic activity for FAL hydrogenation to the target FOL product over the different catalysts ^a^.

Entry	Catalyst	FAL Conversion (%)	FOL Yield <break/>(%)	FOL Selectivity (%)
1	Cu/CaO	38.0 ± 1.3	38.0 ± 1.3	100.0 ± 0.0
2	Co_0.49_Cu_1_/CaO	74.2 ± 4.1	74.2 ± 4.1	100.0 ± 0.0
3	Co_0.96_Cu_1_/CaO	91.8 ± 2.6	89.4 ± 3.4	97.3 ± 1.0
4	Co_1.40_Cu_1_/CaO	100	>99	>99
5	Co_1.94_Cu_1_/CaO	100	>99	>99
6	Co/CaO	9.5 ± 2.1	9.4 ± 1.9	98.7 ± 1.6
7 ^b^	Bulk Co_1.40_Cu_1_	33.5 ± 3.3	33.3± 3.4	99.5 ± 0.4
8 ^c^	Bare CaO	1.6 ± 0.0	1.6 ± 0.0	100.0 ± 0.0
9 ^d^	Bulk Co_1.40_Cu_1_ + bare CaO	15.7 ± 0.9	15.7 ± 0.9	100.0 ± 0.0

^a^ All the experiments were conducted at a reaction temperature of 120 °C, a reaction time of 2 h, initial H_2_ pressure of 20 bar, and catalyst loading of 20% based on 1 g of furfural feedstock, ^b^ The bulk CoCu catalyst was prepared by the combustion of metal nitrate and a subsequent reduction at 500 °C for 3 h in the presence of pure H_2_, ^c^ The bare CaO was obtained by calcination at 900 °C for 5 h in stagnant air, and ^d^ The combination between bulk CoCu and bare CaO was prepared by physical mixing using an equal mass of two components.

**Table 2:** Recent development of non-noble metal catalysts for FAL hydrogenation to FOL in a batch reactor under H_2_ atmosphere.

Entry	Catalyst	Mass Ratio of FAL to Catalyst	Solvent	T	Time	P	FAL Conversion	FOL Yield	Ref.
				°C	h	bar	%	%	
1	NiCoZn@CN-600 ^a^	9.6	THF	160	4	20	100	99	[21]
2	Ni_0.5_@OMC-600 ^b^	2.9	1-Propanol	180	4	30	99	98	[34]
3	PMS-2-CuCo ^c^	9.6	Methanol	110	1	10	99	98	[35]
4	Cu_1_Co_5_ ^d^	100	2-Propanol	180	5	10	100	38.1	[36]
5	CuCo_0.4_/C-873	12.9	Ethanol	140	N/A	30	98.7	96.4	[47]
6	Cu–Co/γ-Al_2_O_3_	4.3	2-Propanol	200	4	40	98.8	25.3	[46]
7	Nano-Co_2_P/Al_2_O_3_	–	Methanol	130	2	40	85	84.2	[37]
8	Na−Cu@TS-1 ^e^	1	2-Propanol	110	2	10	93	91.2	[31]
9	Cu_2_Zn/SiO_2_	7.7	H_2_O	120	4	25	81.9	77.6	[38]
10	CuAl_2_O_4_	–	2-Propanol	170	1	30	100	>95	[39]
11	Cu/C	6	2-Propanol	170	3	20	99.6	98.9	[40]
12	*m-*Co_3_O_4_(*p6 mm*)-350 ^f^	50	2-Propanol	180	3	20	100	65	[41]
13	ZnCo-US@NC-700 ^g^	0.48	Ethanol	120	4	20	100	91.5	[42]
14	Co_1.40_Cu_1_/CaO	3.3	2-Propanol	100	4	20	100	98.9	This work
15	Co_1.40_Cu_1_/CaO	5	2-Propanol	120	2	20	100	>99	This work

^a^ CN = N-doped carbon., ^b^ OMC = ordered mesoporous carbon., ^c^ PMS = porous metal silicate materials., ^d^ The catalyst was used in the form of ordered mesoporous CuCo oxide, ^e^ TS-1 = titanium silicalite-1, ^f^ The mesoporous Co_3_O_4_ catalyst was prepared by a nanocasting method using SBA-15 as a template and further reduced at 350 °C, and ^g^ ZnCo bimetal ZIF was prepared by an ultrasonic-assisted method and calcined under a N_2_ atmosphere at 700 °C.

**Table 3:** Elemental composition and physical properties of the different catalysts.

Sample	Elemental Composition (%) ^a^						S_BET_ ^b^	V_p_ ^c^	D_p_ ^d^
	Co	Cu	Ca	Si	Mg	O	(m^2^ g^–1^)	(cm^3^ g^–1^)	(nm)
Cu/CaO	–	28.5	40.0	2.9	0.6	28.0	3	0.031	59.0
Co_0.49_Cu_1_/CaO	5.4	12.0	47.3	3.7	0.6	31.0	6	0.048	44.1
Co_0.96_Cu_1_/CaO	10.7	12.0	42.4	3.2	0.8	30.9	6	0.059	44.1
Co_1.40_Cu_1_/CaO	15.3	11.8	38.8	2.6	0.6	30.9	10	0.096	37.9
Co_1.94_Cu_1_/CaO	19.8	11.0	35.5	2.3	0.5	30.9	7	0.068	37.9
Co/CaO	25.2	–	39.0	2.5	0.6	32.7	6	0.045	51.1
CaO	–	–	62.2	4.1	1.1	32.6	7	0.097	143.8

^a^ Elemental composition obtained from XRF measurement. ^b^ S_BET_ obtained from the adsorption branch of the N_2_ isotherm. ^c^ V_p_ calculated from N_2_ adsorption at a relative pressure of ~0.95. ^d^ D_p_ obtained from the desorption branch using the BJH method.

## Data Availability

Not applicable.

## Supplementary Materials

The following supporting information can be downloaded at: https://www.mdpi.com/article/10.3390/nano12091578/s1, Table S1: Summary of binding energies of Cu*2p*, Co*2p*, and Ca*2p* and kinetic energies of Cu LMM from the XPS spectra; Figure S1: GC chromatograms of (a) FAL in 2-propanol before the reaction, and liquid product after reaction at 120 °C, H_2_ pressure of 20 bar, and reaction time of 2 h over (b) Co_1.40_Cu_1_/CaO and (c) Co_1.94_ Cu_1_/CaO catalysts; Figure S2: (A) N_2_ adsorption and desorption isotherms, and (B) pore size distribution of (a) CaO, (b) Cu/CaO, (c) Co_0.49_Cu_1_/CaO, (d) Co_0.96_Cu_1_/CaO, (e) Co_1.40_Cu_1_/CaO, (f) Co_1.94_Cu_1_/CaO, and (g) Co/CaO catalysts; Figure S3: H_2_-temperature programed reduction profiles of bulk (a) Cu, (b) Co, and (c) CoCu samples; Figure S4: Representative FE-SEM images of the (a) CaO support, (b) Cu/CaO, (c) Co/CaO, and (d) Co_1.40_Cu_1_/CaO catalysts; Figure S5: Typical TEM image of the bare CaO support.
