# Supplementary material for: Effect of Co-Doping on Cu/CaO Catalysts for Selective Furfural Hydrogenation into Furfuryl Alcohol
Source: Nanomaterials (Basel). 2022 May 6;12(9):1578. doi: 10.3390/nano12091578 (PMC9102403; doi:10.3390/nano12091578)
Supplement: Supplementary file 1 [file nanomaterials-12-01578-s001.zip › nanomaterials-1706829-supplementary-1.pdf]

Supplementary Information

Effect of Co-Doping on Cu/CaO Catalysts for Selective Furfural Hydrogenation into Furfuryl Alcohol

Munsuree Kalong [1], Sakhon Ratchahat [1], Pongtanawat Khemthong [2], Suttichai Assabumrungrat [3,4] and Atthapon Srifa [1,*]

[1] Department of Chemical Engineering, Faculty of Engineering, Mahidol University, Nakhon Pathom 73170, Thailand; kalong.munsuree@gmail.com (M.K.); sakhon.rat@mahidol.edu (S.R.)
[2] National Nanotechnology Center (NANOTEC), National Science and Technology Development Agency (NSTDA), Pathum Thani 12120, Thailand; pongtanawat@nanotec.or.th
[3] Center of Excellence in Catalysis and Catalytic Reaction Engineering, Department of Chemical Engineering, Faculty of Engineering, Chulalongkorn University, Bangkok 10330, Thailand; suttichai.a@chula.ac.th
[4] Bio-Circular-Green-Economy Technology & Engineering Center (BCGeTEC), Department of Chemical Engineering, Faculty of Engineering, Chulalongkorn University, Bangkok 10330, Thailand
* Correspondence: atthapon.sri@mahidol.edu

Citation: Kalong, M.; Ratchahat, S.; Khemthong, P.; Assabumrungrat, S.; Srifa, A. Effect of Co Doping on Cu/CaO Catalysts for Selective Furfural Hydrogenation into Furfuryl Alcohol. *Nanomaterials* 2022 12(9), 1578 https://doi.org/10.3390/nano12091578

Academic Editor(s): Alexandru Mihai Grumezescu

Received: 15 April 2022
Accepted: 4 May 2022
Published: date

Publisher's Note: MDPI stays neutral with regard to jurisdictional claims in published maps and institutional affiliations.

Copyright: © 2022 by the authors. Submitted for possible open access publication under the terms and conditions of the Creative Commons Attribution (CC BY) license (https://creativecommons.org/licenses/by/4.0/).

Table S1. Summary of binding energies of Cu*2p*, Co*2p*, and Ca*2p* and kinetic energies of Cu LMM from the XPS spectra.

Catalyst	Cu*2p* Binding energy (eV)			Co*2p* Binding energy (eV)				Ca*2p* Binding energy (eV)	Cu LMM Auger Kinetic energy (eV)		
	Cu^{+}/Cu^{0}	Cu^{2+}	Sat.	Co^{0}	Co^{3+}	Co^{2+}	Sat.	Ca^{2+}	Cu^{0}	Cu^{2+}	Cu^{+}
Cu/Cao	932.5<br>952.3	934.5<br>954.0	943.0<br>963.7	-	-	-	-	347.0<br>350.5	918.7	917.6	916.5
$Co_{1.40}Cu_{1}/CaO$	932.4<br>952.0	934.3<br>954.3	942.8<br>963.0	779.2<br>794.3	780.5<br>795.8	781.4<br>797.3	785.2<br>802.9	347.3<br>350.7	918.7	917.6	916.5
Co/CaO	-	-	-	779.2<br>794.3	780.5<br>795.8	781.4<br>797.3	785.2<br>802.9	347.1<br>350.6	-	-	-

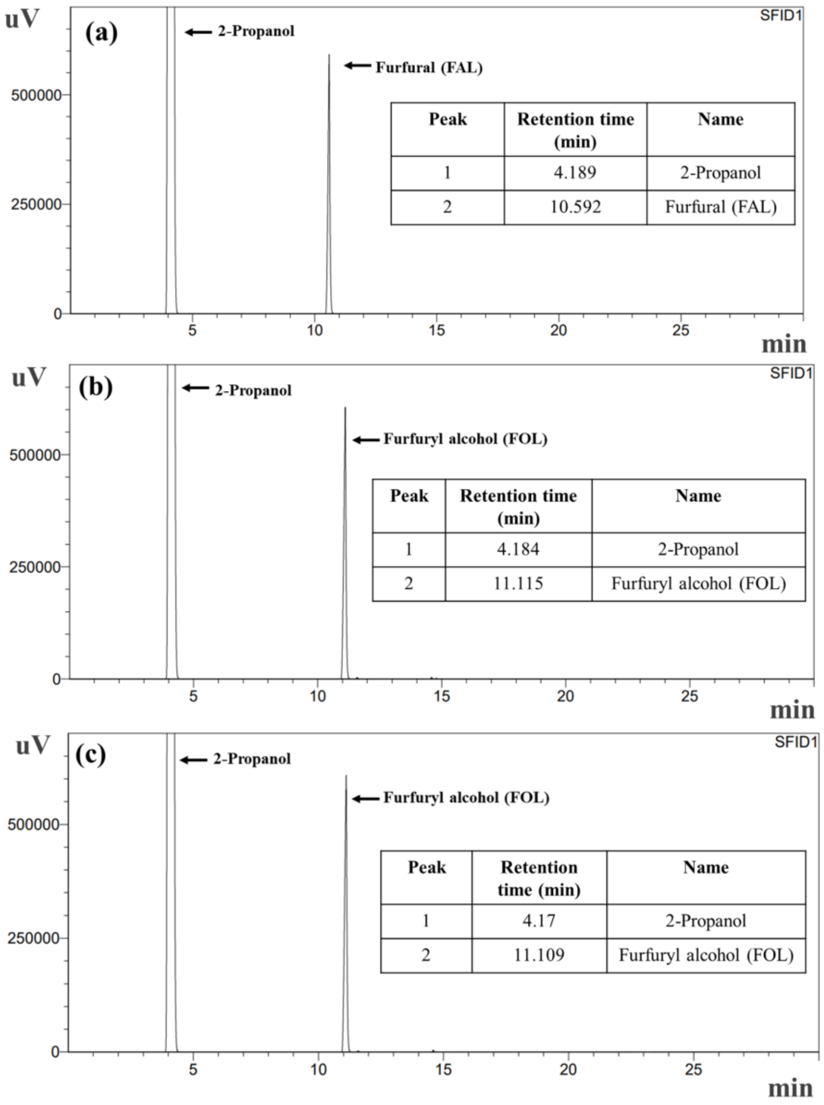

Figure S1. GC chromatograms of (a) FAL in 2-propanol before the reaction, and liquid product after reaction at 120ºC, H_2 pressure of 20 bar, and reaction time of 2 h over (b) $Co_{1.40}Cu_1/CaO$ and (c) $Co_{1.94}Cu_1/CaO$ catalysts.

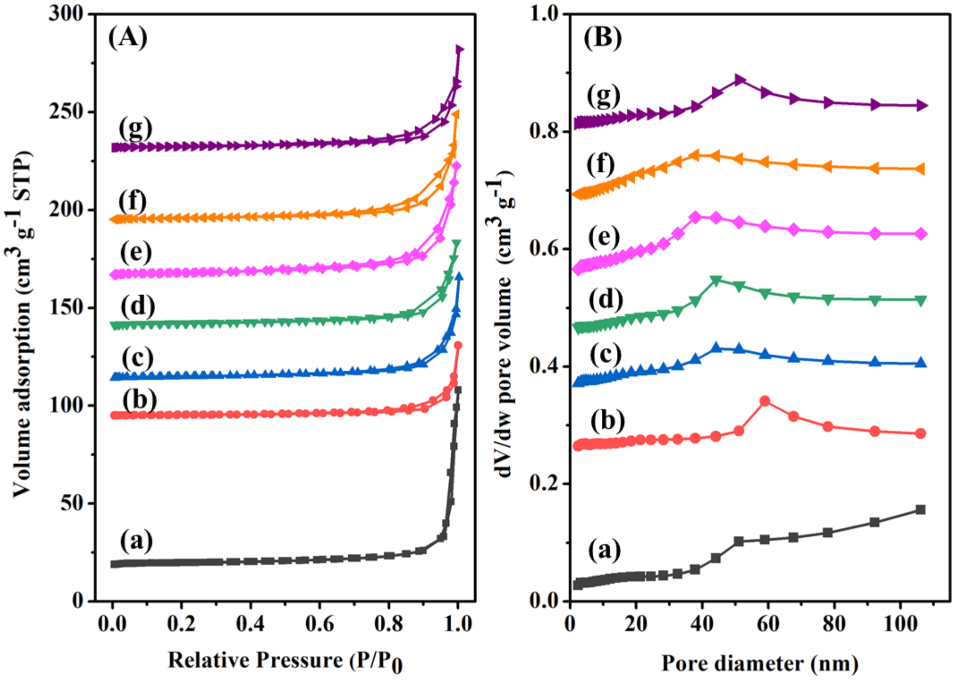

Figure S2. (A) N_2 adsorption and desorption isotherms, and (B) pore size distribution of (a) CaO, (b) Cu/CaO, (c) $Co_{0.49}Cu_1$/CaO, (d) $Co_{0.96}Cu_1$/CaO, (e) $Co_{1.40}Cu_1$/CaO, (f) $Co_{1.94}Cu_1$/CaO, and (g) Co/CaO catalysts.

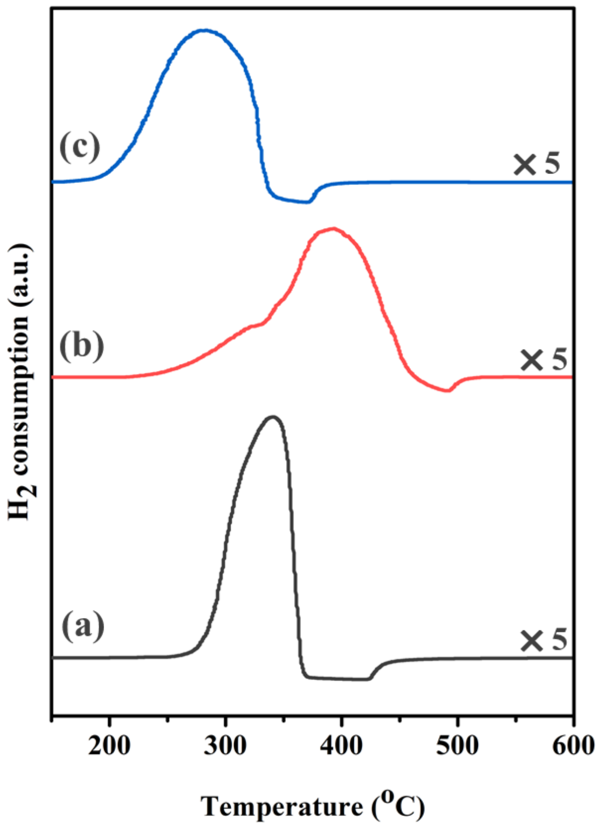

Figure S3. H_2-temperature programed reduction profiles of bulk (a) Cu, (b) Co, and (c) CoCu samples.

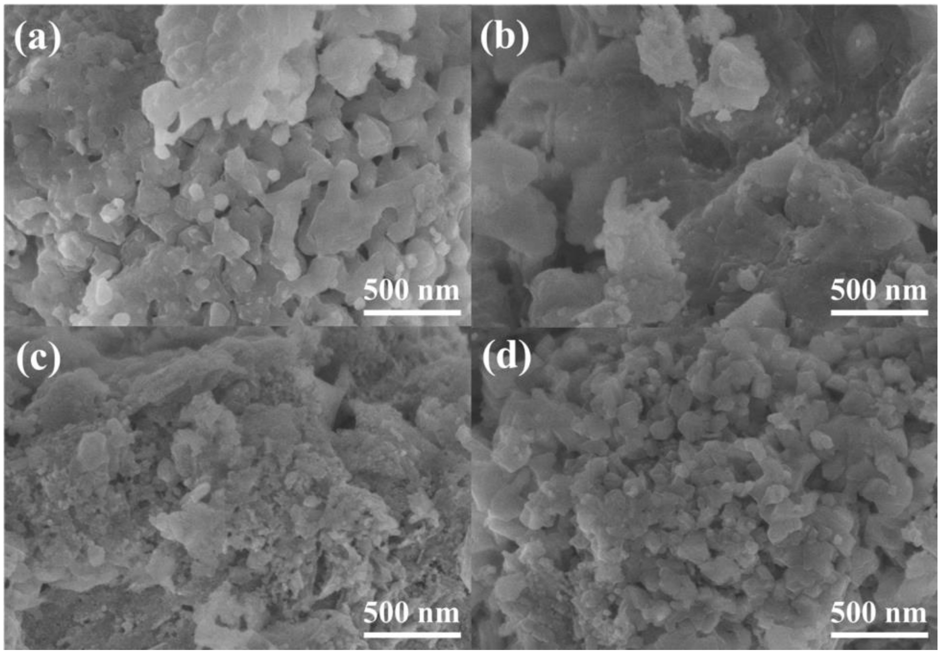

Figure S4. Representative FE-SEM images of the (a) CaO support, (b) Cu/CaO, (c) Co/CaO, and (d) $Co_{1.40}Cu_1$/CaO catalysts.

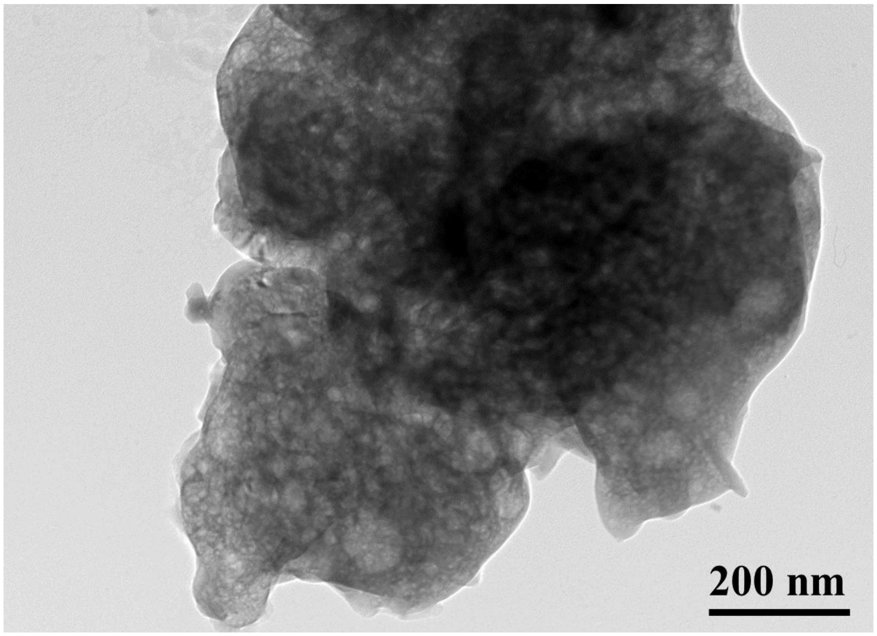

Figure S5. Typical TEM image of the bare CaO support.
